# The Microbiota of Grana Padano Cheese. A Review

**DOI:** 10.3390/foods10112632

**Published:** 2021-10-29

**Authors:** Giorgio Giraffa

**Affiliations:** Council for Agricultural Research and Economics, Research Centre for Animal Production and Aquaculture (CREA-ZA), Via Lombardo 11, 26900 Lodi, Italy; giorgio.giraffa@crea.gov.it; Tel.: +39-0371-45011

**Keywords:** Grana Padano cheese, cheese microbiota, undefined starters, starter lactic acid bacteria (SLAB), non-starter lactic acid bacteria (NSLAB), core microbiota, pan microbiota, microbial selection, microbial dynamics

## Abstract

Grana Padano (GP) is the most appreciated and marketed cheese with Protected Designation of Origin in the world. The use of raw milk, the addition of undefined cultures (defined as ‘sieroinnesto naturale’), the peculiar manufacturing proces, and the long ripening make the cheese microbiota play a decisive role in defining the quality and the organoleptic properties of the product. The knowledge on the microbial diversity associated with GP has been the subject, in recent years, of several studies aimed at understanding its composition and characteristics in order, on the one hand, to improve its technological performances and, on the other hand, to indirectly enhance the nutritional quality of the product. This review aims to briefly illustrate the main available knowledge on the composition and properties of the GP microbiota, inferred from dozens of studies carried out by both classical microbiology techniques and metagenomic analysis. The paper will essentially, but not exclusively, be focused on the lactic acid bacteria (LAB) derived from starter (SLAB) and the non-starter bacteria, both lactic (NSLAB) and non-lactic, of milk origin.

## 1. Introduction

The specific and highly appreciated characteristics of cheeses, including PDO cheeses, are the result of the interaction of several factors such as the quality of raw milk, the farming methods and processing technology which, in many cases, involves the use of undefined microbial cultures. These elements can in turn contribute to modulate, both qualitatively and quantitatively, the microbial composition of the mature product, giving it a specific microbial imprint. Moreover, the microbiota plays an invaluable role in the transformation and ripening of cheeses. The microorganisms present in milk, together with those added with the starter and the environmental contaminants represent, with different dynamics and relationships, the engine of the biochemical transformations that define the quality of the products [1,2]. Grana Padano (GP), the most commercialized and appreciated PDO cheese in the world, is not an exception to this pattern.

With a production size of around 210,000 tons in 2020 (over 5,200,000 cheeses) and an ever-expanding export (40% of total production, +3.3% compared to 2019), GP is among the most known, consumed, and appreciated Italian cheeses in the world. The first available data recorded in the last 12 months refer to a sharp slowdown in the performance of GP, especially in terms of exports, due to the pandemic event, which, however, affected the entire dairy sector (https://www.granapadano.it (accessed on 9 September 2021)). Despite this, GP remains the main PDO in the national dairy sector. Grana Padano is a hard cooked cheese, produced by adding undefined starters (the so-called ‘sieroinnesto naturale’) to raw, partially skimmed milk. The complex cheese microbiota, with its enzymatic load and dynamics, plays a fundamental role in carrying out the biochemical and structural changes that characterize the long ripening (up to 18–24 months) of the product [3].

The scientific literature regarding the composition and microbial dynamics of GP and similar cheeses is very extensive and constantly evolving. Two exhaustive review articles described the properties and characteristics of the microbiota of GP and similar PDO cheeses (e.g., Parmigiano Reggiano, PR) during ripening [3,4]. In this regard, extensive knowledge is accumulating thanks also to advances in molecular investigation techniques (metagenomics, metabolomics, metatranscriptomics), which are offering an increasingly complete picture of the microbial community associated to cheese [4,5,6]. The purpose of this paper is to provide a concise but complete overview of the extensive literature available on the microbiota of GP, focusing the analysis on the relatively most recent (last 15–20 years) studies. The review will mainly concern microorganisms deemed useful (e.g., especially the lactic acid bacteria, LAB); however, the unwanted microbiota (especially clostridial spoilers), which still cause commercial depreciation of the product, will also be mentioned.

## 2. Origin of the Microbiota

### 2.1. Microorganisms from Raw Milk

**Figure 1:**
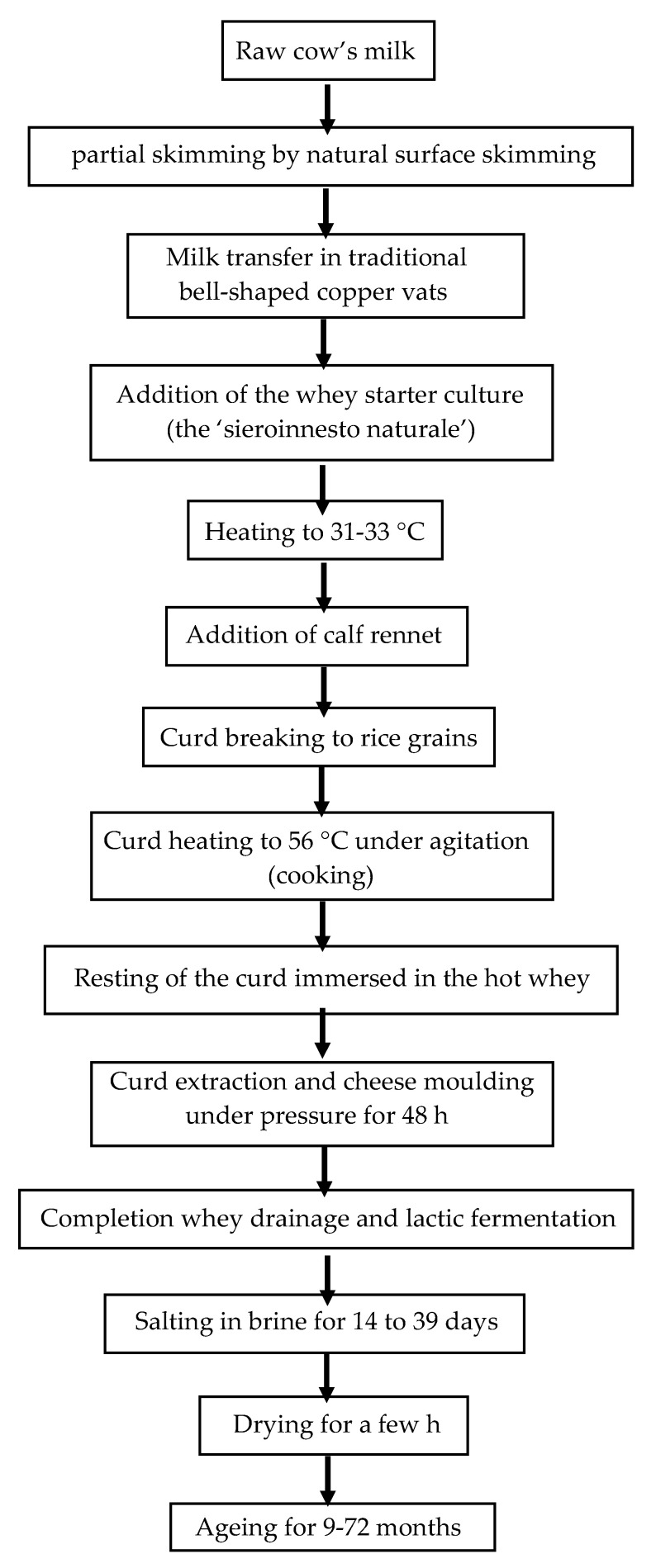
Flow diagram of the Grana Padano cheese production.

Grana Padano cheese is produced in about a hundred dairies located in a very extensive production area within the Po Valley. Although the methods and criteria for cattle breeding are substantially similar, the microbial content of the raw milk destined to GP cheesemaking can be considerably affected by differences in farm management practices and changes in relation to seasonal, climatic, and environmental variations. The microorganisms present in raw milk then undergo the action of some key technological phases of GP cheese manufacturing (spontaneous creaming of milk, whey starter addition, curd heating at 53–56 °C, prolonged stasis under the hot curd whey, long whey drainage, very long ripening), which address their selection and dynamics. Figure 1 presents a flow chart summarizing the main steps of GP cheese production process. In this framework, the composition of the raw material is the starting point that probably will give a peculiar imprint to the final composition of the cheese [2,3]. In the current state of knowledge, few studies have been carried out on the microbial content of milk for GP cheese manufacturing. Indeed, most of the papers report the microbial composition of the typical starter culture (the ‘sieroinnesto naturale’-SN) used for GP production and the dynamics that arise during cheese manufacturing and ripening, with little or no emphasis on their connection to the raw milk composition.

Bovine milk always contains a significant amount of LAB, with considerably wide quantitative ranges, belonging to the genera *Lactococcus* (1–4 log_10_ cfu/mL), *Streptococcus* (1–4 log_10_ cfu/mL), *Lactobacillus* (2–4 log_10_ cfu/mL), and *Leuconostoc* (2–3 log_10_ cfu/mL) [7]. Santarelli et al. [8] found mesophilic LAB ranging between 10^4^ and 10^5^ cfu/mL in raw milk samples for GP cheese. After culture enrichment, the samples were analyzed by length-heterogeneity-PCR (LH-PCR) and, after 16 rRNA gene sequencing of isolates, resulted composed, in decreasing order, of *Streptococcus* (*S.*) *uberis*, *Lactococcus* (*Lc.*) *lactis* subsp. *lactis* and subsp. *cremoris*, *Lactobacillus* (*L.*) *delbrueckii* subsp. *lactis*, *Leuconostoc* (*Ln.*) *mesenteroides* subsp. *mesenteroides*., *Enterococcus* (*E.*) *faecalis*, *E. faecium*, *L. helveticus*, *Lentilactobacillus* (*Lent.*) *hilgardii*, *Limosilactobacillus* (*Lim.*) *fermentum*, *L. gasseri*, and *Lacticaseibacillus* (*Lcb.*) *rhamnosus* were also, although less frequently, identified. Meaningfully, LH-PCR revealed that most of the microbial species found later (in both SN and cheese) may predominantly originate from the milk used for GP cheese manufacturing [4,8]. 

According to the production specification for GP, refrigeration of milk at farm level is allowed, as long as the temperature is not <8 °C, even during transport to processing sites [3]. This operation is essential for the success of the product. It has a dual purpose: (i) to favor the development of useful lactic microbiota, belonging to the group of non-starter LAB (NSLAB), and (ii) to contain that of pathogenic or spoilage microbes (psychrotrophs, spore-forming). The next step, i.e., the spontaneous creaming, leads to the so-called ‘cleaning effect’. During this step, which is performed at the dairy between 8 and 20 °C before cheese manufacture to obtain half-skimmed milk, most of bacteria, especially the spore-formers, are dragged upwards by the spontaneous flotation of fat globules and removed with the cream [3]. These broad temperature range can have a noticeable influence on the outcome of the microbiota of skimmed milk. In a study on the influence of different time-temperature combinations on the microbial composition of raw cows’ milk used for ‘Grana Trentino’ cheese-making, several microbial groups (psichrotrophic bacteria, coliforms, mesophilic and thermophilic LAB, pseudomonads, and clostridia) were identified in vat milk after creaming. More specifically, mesophilic and thermophilic LAB ranged between 2.7–3.9 and 2.2–2.9 log_10_ cfu/mL, respectively, butyric clostridia were not detectable (<2 log_10_ cfu/mL), and psychrotrophic bacteria did not exceed 3.2 log_10_ cfu/mL [9]. The combined effect of the progressive microbial impoverishment of milk, pursued thanks also to the improvement of hygienic production and storage practices, combined with the cleaning effect of milk creaming which, not being selective, also reduces the number of pro-technological microbes, has inevitably led the need of using starter cultures.

### 2.2. The ‘Sieroinnesto Naturale’ (SN) 

The SN is daily prepared by cheese makers from a portion of cooked, unacidified whey, which is collected from cheese vat at the end of the curd heating step, carried out at 53–56 °C for 30–70 min. Unacidified collected whey is held under a gradient of temperature or left spontaneously to cool for 18–24 h. More equipped dairies can use special fermenters in which a given temperature (generally 42–45 °C) is set, extending the incubation until an acidity of 55–65° SH/100 (pH 3.3–3.6) is reached. Regardless on the composition of the raw milk, the above steps reduce the microbial diversity, inducing a selection of few moderately heat-tolerant, aciduric, and thermophilic LAB species and strains, which reach 8–9 log cfu/mL and become dominant in the ready-to-use SNs [3,8,10,11]. The extensive scientific literature available to date agrees that SN cultures for GP, but also for PR, are characterized by a limited number of LAB species (or subspecies) belonging to *L. helveticus* (the most common one), *L. delbrueckii* subsp. *lactis*, *Lim. fermentum*, and *S. thermophilus* [3,8,10,11,12,13,14]. In this regard, it would be appropriate to forsake the term ‘undefined’ associated with the thermophilic starters for GP (or PR) which, due to the peculiarities that characterize their preparation, instead show a generally well-defined and quite invariable species composition from dairy to dairy, with only small variations in the relative abundance and number of strains. Consequently, it would be also desirable to replace the term ‘natural whey starters’ with a more appropriate definition, which should take into consideration the methods of preparation and the consequent selection of the microbiota of SN. ‘Spontaneously selected, whey cultures’ is the new proposed term for SN.

Early studies on the composition of the SNs have often reported the finding of *L. delbrueckii* subsp. *bulgaricus*, while in recent years there has been convergence in stating that only the subsp. ‘lactis’ is present in these cultures, although both subspecies were isolated from milk for GP and PR cheeses [4]. Even though the two microorganisms are both thermophilic and acidophilic, exhibit identical thermal resistance and proteolytic pattern, genetic and phenotypic differences, alongside the improvement and refinement of microbial identification techniques, may explain the prevalence of the ‘lactis’ subspecies. Indeed, *L. delbrueckii* subsp. *lactis* shows a wider carbohydrate fermentation pattern and, especially, a lactose-PTS system [15], which makes it able to metabolize the residual galactose in cooked, unacidified whey. Additionally, amino acid (AA) biosynthesis capacities are more severely reduced in the ssp. *bulgaricus* than in the ssp. *lactis* [15], which make the latter less dependent on an easily assimilable, external nitrogen source, especially free AAs, of which cooked whey is relatively lacking. It could be speculated that both factors may favor a more rapid and efficient growth of the subspecies “lactis” to the detriment of the subspecies “bulgaricus” in both NS and cheese. It is conceivable that *L. delbrueckii* subsp. *bulgaricus*, although is a species frequently isolated from dairy products (e.g., fermented milks and cheeses), does not seem to adapt to the environment of GP and similar cheeses. Less frequent (subdominant) species, such as *Lcb. casei/paracasei*, *Lactiplantibacillus* (*Lpb.*) *plantarum* or *Levilactobacillus* (*Lev.*) *brevis*, have sometimes been reported. Both dominant and subdominant taxa of SN are present with different abundance and respective ratios in relation to ecological and technological factors and the different methods for their identification and quantification, which include both molecular and culture-dependent techniques [4,16,17,18]. In this regard, Fornasari et al. [19] underlined the importance of restoring the cultivability of acid stressed *S. thermophilus* which, after recovery, was quantified at considerable numbers (10^7^–10^8^ cfu/mL) in SN for GP. Such unexpectedly high levels of *S. thermophilus* showed how likely this bacterium, and eventual other LAB species, could be under-quantified in SNs without first restoring their viability and cultivability.

Minimal but sometimes significant species fluctuations linked to the initial composition of the whey at the end of processing (that in turn reflects the microbial composition of the starting milk) or to dairy-specific variations in the preparation of the cultures, have also been reported. Time-temperature combinations and the final acidity of the cultures, which can be very variable between dairies, are among the most important players in microbial selection. Correlations between species abundance and final SN acidity, which induces specific microbial dynamics during the preparation of the culture, have been reported. For example, *L. helveticus* and *Lim. fermentum* are positively correlated with acidity while *L. delbrueckii* and *S. thermophilus* show an opposite trend [18,20]. Conversely, an increase of the relative abundance of *L. delbrueckii* and a concomitant decrease of *L. helveticus*, have been observed in cooked, unacidified whey for PR [20]. In factories where the preparation of the SN occurs by spontaneous cooling, the production cycle of the SN can be divided into two phases: ‘thermophilic’, when the temperature is between 55–40 °C and ‘mesophilic’, when the temperature is in the range 40–20 °C. In a recent paper on SN for Trentingrana, it was hypothesized that a different length of the mesophilic phase, applied in some factories, would explain the unexpected finding and dominance (>30% of isolates) of *Lev. brevis*. In the same paper, authors showed that the frequency of the LAB strains into the different cultures is mainly dairy-specific rather than dependent on the month of production [17]. 

An often, underestimated problem in undefined cultures, including SN, is the presence of bacteriophages, which regulate dynamics and alternation between strains within the starter. Numerous studies describe the detection and quantification of bacteriophages isolated from NS for GP cheese and active vs. *L. helveticus* and *L. delbrueckii* subsp. *lactis* [17,21,22,23]. It should be noted that, apparently, only in rare cases the presence of bacteriophages interferes with the technological performance of the SN, which reacts to the phage attack thanks to the establishment of a co-evolutionary mechanisms between parasite and host. It has been attributed to phages an ecological role in selecting, among the different populations, phage-resistant strains able to counteract the loss of the sensitive ones, thus preserving the overall technological performances of the culture. This was partly confirmed by the concomitant finding in the same culture of different *L. helveticus* sub-populations of both phage-sensitive and phage-resistant strains, these latter rapidly becoming dominant over the whole population and, in turn, being inhibited by the respective phages according to the well-known kill-the-winner concept [24]. This co-evolutionary mechanism between host and parasite spontaneously perpetuates over time, activating a sort of rotation between strains similar to that applied by the industry to defined starters, which preserves the overall technological performance of the cultures [1,2,17,25]. 

## 3. Microbial Selection during Cheese Processing and Molding 

The microbiota of raw milk, together with that transferred to the vat milk with the SN, undergoes a qualitative-quantitative selection and evolution during the early steps of cheese manufacture. This selection is the consequence of several concomitant actions, related mainly to the time-temperature parameters during the cooking of the curd (53–56 °C for 30–70 min), the conditions of curd sedimentation under hot whey before extraction from the vat (which can last between 40 and 70 min), and those of whey drainage of the molded curd. The interplay between these factors, especially the wide range of the possible time-temperature combinations, influences the selection of the starter microorganisms, with little or no influence on their growth and curd acidification. To this regard, the decrease in the lactose content in vat does not seem to be attributed to lactic acid fermentation, as indicated by the small increase in lactic acid and galactose contents at molding [3]. It is above all the thermal stress induced by cooking that selects the growth and dynamics of the species during the maintenance of the curd under hot whey and, subsequently, during the preparation of the SN [14]. As aforementioned, meaningful in this regard is the higher incidence of *L. delbrueckii* compared to *L. helveticus* in cooked, hot, and not acidified whey [20]. To limit this drawback, some factories equipped with fermenters prefer to accelerate the cooling of the hot whey from the cooking temperature to 48 °C (or even lower) to reduce the heat stress and promote, in addition to the thermophilic homofermentative lactobacilli, the growth of *S. thermophilus* [3].

In molded cheeses, other selective factors come into play. In extra sized cheeses such as GP, where the cooking temperatures are high, significant thermal gradients between the external and internal curd layers (up to 15 °C within 7 h from molding) may long persist, influencing the microbial growth across the cheese [3,14]. In a pioneering study on grana-like cheeses, Giraffa et al. [26] showed that the microbial growth of thermophilic LAB during molding was maximum between 0 and 6 h in the cheese exterior and between 6 and 24 h in the cheese core. This variation occurred because the inner layers are still around 52 °C 6 h after molding, which is relatively far from the *optimum* for the growth of thermophilic LAB. In addition, LABs are affected by heat stress, which is more intense in the cheese interior. This gap seems to persist later, during cheese ripening, as shown by Monfredini et al. [27] who found higher LAB counts in the outer areas of 9–18 months aged GP cheese, compared with those in the inner ones. This thermal gradient has a direct effect on the distribution in species across the cheese. Specifically, *L. helveticus* prevails in the external areas while the distribution of *L. delbrueckii* and heterofermentative lactobacilli, which increase slowly from the cheese molding up to 48 h, is more variable in the internal areas than in the external ones. *L. fermentum* was also isolated from curd between 6 and 48 h from molding [26]. 

## 4. Microbiology of Cheese

The microbiota of the mature GP can vary significantly between different cheeses. This may be due to the heterogeneous microbiological composition (both qualitative and quantitative, especially in terms of minor species and single strains) of the raw milk and SN, according to seasonal or geographical differences and to minimal, but significant, variations in the milk management before coagulation and the physical-chemical conditions of the production environments, especially the thermal-hygrometry of the ripening rooms [3]. To this regard, the extensive scientific literature on the microbiology of GP and similar cheeses, such as PR, unanimously reports the presence of a ‘core’ microbiota, composed of a relatively small number of dominant species of LAB and a ‘pan’ microbiota, which characterizes individual cheeses within the large production area. The selection of the core microbiota derives mainly from the modulating action of the shared cheese technology and the use of SN, which is prepared with similar methods across dairies and contains, as already mentioned, a limited number of species [3,8,10,12,13,14]. The pan microbiota, on the other hand, derives from raw milk and environmental contamination and is therefore a mirror of the company’s variability in the composition of milk, the microbial presence in the processing and maturation environments and, only to a lesser extent, any minor dairy-specific variations in technology and in the composition of the SN [4].

### 4.1. Core and Pan Microbiota

Dozens of papers and review articles carried out in the last twenty years converge almost uniquely in identifying a core microbiota in GP and similar cheeses (e.g., PR), consisting of a limited number of LAB species belonging, with minor qualitative and quantitative inter-dairy and sample variations, to *L. delbrueckii*, *L. helveticus*, *Lcb. rhamnosus*, *Lcb. casei*, *Lcb. paracasei*, *Lim. fermentum*, *S. thermophilus* and, less frequently, to *Ped. acidilactici Lc. lactis*, and *Lpb. plantarum* [3,4,8,12,14,28,29,30,31]. A very recent investigation carried out on 118 samples covering all the GP production area included, within the core microbiota, also *Lc. raffinolactis* [31]. The dominance of the above LAB species within the core microbiota largely reflects that of the SNs used in the production of GP and, to a lesser extent, that of the NSLAB species typically found in long-ripened cheeses [1]. Notably, in cheeses produced in different geographical locations and ripening times, the composition of the core microbiota appears quite stable over time, despite the selective action of the lysozyme (admitted as an anticlostridial agent in GP cheeses) and the application of very different methods of sampling, microbial identification (culture dependent or DNA/RNA-based), and quantification.

As stated above, the composition of the pan microbiota of GP is very heterogeneous and, being strictly related to the microbial composition of raw milk, more widely variable than the core microbiota. External contamination sources including tanks, cheese vats, benches, cloths, knives, and other factory tools, may further contribute to ‘inoculate’ bacteria into milk and on cheese during GP processing and ripening [3,4]. The pan microbiota includes both LAB (*Lactobacillus*, *Lactococcus*, *Leuconostoc, Streptococcus, Lentilactobacillus*) and non-LAB (e.g., *Propionibacterium*, *Acinetobacter*, *Pseudomonas*, *Macrococcus*, *Staphylococcus*, enterobacteria) genera [8,14,21,29,30,31,32]. At the species level, LABs of probiotic or functional interest (*Schleiferlactobacillus harbinensis*, *L. jensenii*, *L gasseri*, *L. johnsonii*) have often been found in GP and PR [31,32,33,34], highlighting these cheeses as a source of additional healthy bacteria, along with the well-known probiotic species (*Lcb. casei*, *Lcb. paracasei*, and *Lcb. rhamnosus*) belonging to the core microbiota of the ripened cheese. The presence of LAB species, such as *Lent. parabuchneri*, *Lent. parafarraginis*, *Lent. hilgardii*, *Lent. diolivorans*, and *L. nasuensis*, used as silage starters or isolated from silage, can be related to corn silage fed to cows producing milk for GP [30,31,35]. The less and less occasional presence and significance of contaminants (such as *Propionibacteria*, *Pseudomonas*, *Acinetobacter*, and *Enterobacteriaceae*) with a potentially negative impact on the quality of cheeses (spoilage, pathogens) will be discussed later (Section 4.3). However, the emerging of an ever-increasing number of bacterial genera and species in GP depends on the frequent applications of DNA (or RNA)-based metagenomic techniques for the study of food-associated microbial communities. The advantage of these techniques is to allow the composition of even the minority microbiota in complex ecosystems, such as cheeses, to be known in an increasingly in-depth way. The limits derive from the fact that, investigating subdominant populations, the taxa are highly dispersed (and very variable, both qualitatively and quantitatively) among different cheese samples, as they are affected by the current wide choice of different methods and pipelines used for metagenomic analysis and data processing [36]. Additionally, no information on cell viability is provided when metagenomics is applied to total DNA. However, the subdivision into core and pan-microbiota is purely didactic since, following the evolution of metagenomics techniques and changes in the microbial composition of raw milk and in cheese production, the boundaries between the two microbial groups are increasingly faint. 

### 4.2. Microbial Dynamics during Ripening

In cheeses with medium-long ripening (>3–6 months), especially those obtained from raw milk, it is generally assumed that starter LAB (SLAB) play a key role in the early stages of cheese production while the non-starter LAB (NSLAB), which can use other carbon sources in addition to lactose and are generally more resistant to osmotic stress, tend to dominate in aged cheeses [5]. GP cheese is not exempt from this general trend. The increasingly harsher conditions taking place with the progressing of the acidification during molding and early ripening (lactose depletion, pH, aw, and redox lowering, curd temperature decreasing, lack of nutrients) leads to a slowing down of the growth and to the autolysis of thermophilic SLAB introduced with the SN, which are progressively replaced by the mesophilic NSLAB predominantly coming from the raw milk and, to a lesser extent, the factory environment, even if this latter aspect would require more specific studies [4,8,37,38,39]. A precise microbial dynamic and shift of the bacterial species which characterize the microbiota of the GP during ripening have been shown in several studies, carried out by both culture-dependent and culture-independent methods. Although in sharp decline during cheese brining, the SLAB belonging to *L. helveticus* and *L. delbrueckii* subsp. *lactis* dominate the GP microbiota at the beginning (1–2 months) of ripening, with prevalence and surviving of the latter even later. With the progressing of the ripening, cell autolysis of SLAB, and the subsequent release of nutrients, is suggested to stimulate the outgrowth of NSLAB mostly belonging to *Lcb. casei*, *Lcb. paracasei*, and *Lcb. rhamnosus*, and, to a lesser extent, to *Pediococcus* spp. (especially the species ‘acidilactici’ and ‘pentosaceous’). *Lim. fermentum, S. thermophilus*, and lactococci (the first two species from the SN) were found, although less frequently, in GP cheese; however, their contribution to cheese ripening has not been fully clarified [3,4,8,14,38,39]. The isolation of *Lim. fermentum* from PR after 12 months of ripening would suggest a high resistance to lysis of this microorganism [33], indirectly confirming a possible role even in GP cheese. *Lim. fermentum*, an obligate heterofermentative, has been isolated from GP curd between 6 and 48 h from production, while high amounts of lysed cells of this microorganism were detected in cheese after two months of ripening [8,26]. *Lim fermentum* would contribute to generate the typical (although less and less frequent) micro holes in the mature product. The same role could be speculated for the obligate hetero-fermenter *Lent. parabuchneri*, recently found within the dominant species in GP and Grana Trentino cheeses [30,31,32]. 

**Figure 2:**
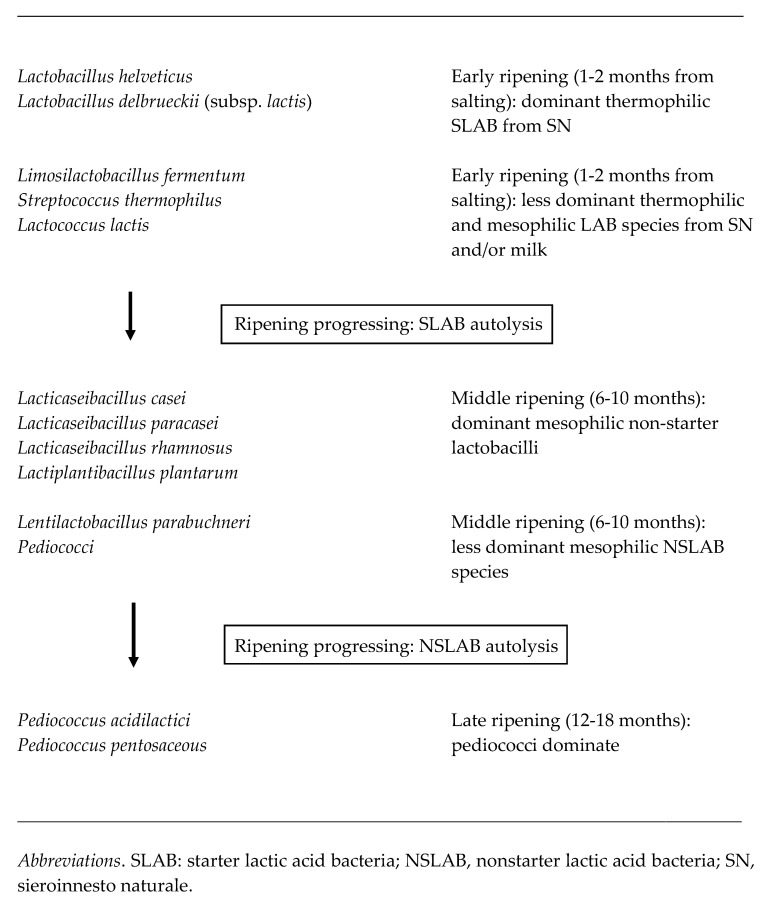
Microbial dynamics of dominant lactic acid bacteria taxa during Grana Padano cheese ripening.

Differently from SLAB, which contribute to cheese ripening by releasing intracellular enzymes mostly in the early ageing phase, NSLAB can multiply after salting and release proteolytic enzymes and flavor compounds into the cheese in more advanced stages of ripening. In this regard, the literature data do not show a clear-cut alternation between the most frequently found, non-starter *Lactobacillus* species (*Lcb. casei*, *Lcb. paracasei*, *Lcb. rhamnosus*, and *Lpb. plantarum*), which can be explained by a variable resistance to the hostile cheese conditions during ripening and a different, strain-specific, propensity to autolysis. Pediococci tend to dominate still alive in the last period of ripening where, after autolysis, contribute to the accumulation of aromatic volatile compounds [3,4,8,28,30,31,37,38,39]. An alternation between *Lcb. rhamnosus* strains isolated from PR revealed how different biotypes with variable adaptability to hostile conditions can dominate in specific stages of ripening [40]. The detection of strains characterizing different ripening times would suggest they may play peculiar roles in defining the final cheese quality. The same phenomenon could also be hypothesized for GP cheese. Additionally, the presence in mature GP cheese of numerous biotypes belonging to species, such *Lcb. casei*, *Lcb. paracasei*, and *Lcb. rhamnosus*, with well-known health-promoting effects and still detectable at quite high levels (10^4^–10^6^ cfu/g) in mature cheeses, implies the need for their in-depth technological and functional characterization. Figure 2 summarizes the microbial dynamics of dominant LAB during GP cheese ripening.

### 4.3. Spoilage and Safety Aspects

In recent years the application of culture-independent techniques and metagenomic analysis for the evaluation of microbial diversity in foods (including cheeses) highlighted the presence of many contaminants, some of which belonging to potentially pathogenic microbial groups, or opportunistic pathogens, even in mature GP. Many of them belong to *Enterobacteriaceae*, *Acinetobacter* spp., and, less frequently, to *Enterococcus* spp., *S. uberis*, *Lc. garviae*, coagulase-negative staphylococci, and cutaneous propionibacteria (e.g., *Propionibacterium acnes*) [14,29,30,31,32]. However, a clear distinction must be made between simple detection and microbial activity (and therefore an expression of pathogenicity or virulence). The presence of undesirable bacteria, such as *Enterobacteriaceae*, *Acinetobacter* spp., *Pseudomonas* spp., or *Psychrobacter* spp., detected in GP and similar cheese by metagenomics, is not surprising as, being members of the resident microbiota of food processing plants, they could still residual not alive in cheese [30,31]. However, the finding of unwanted microorganisms through metagenomics does not necessarily imply that the cells are in a state of vitality. In general, the culture-independent systems are based on the analysis and amplification of the total DNA extracted from the matrix, which do not give any information on the cell metabolic activity. There are few studies in which the state of viability of microbial contaminants isolated from GP has been evaluated. In a metagenomic analysis on Grana-like cheese, Alessandria et al. [29] highlighted that *Prop. acnes*, which appeared as the main contaminant in milk, remained metabolically active until the end of ripening.

There are no epidemiological data regarding possible infections or food poisoning of microbial origin due to the consumption of GP cheese and similar cheeses. The production and ripening of this cheese can be considered a perfect synthesis of the ‘hurdle tools’ concept, which can be defined as an approach combining several mitigating barriers or obstacles (decrease in microbial load by spontaneous milk creaming, use of very acidifying and active starter cultures, extended curd cooking at 53–56 °C, extended stasis of cooked curd before molding, prolonged brining, very long ripening time leading a sharp A_w_ decrease, the formation of the rind, and the drying of the cheese), each of which alone would be insufficient to control or even eliminate pathogens in food products. The studies carried out so far to evaluate the potential development of pathogenic microorganisms, voluntarily added to milk, along the processing of GP cheese, have confirmed that the storage conditions of raw milk and those of processing and ripening allow for the control of pathogens accidentally present along the cheese production chain. A laboratory-scale challenge test to evaluate the fate of pathogenic bacteria during the spontaneous creaming process showed that no growth of *Listeria monocytogenes*, *Salmonella* spp., *Escherichia coli* O157:H7, or *Staphylococcus aureus* occurred within 16 h at 8 and 15 °C. The study indicated that avoiding a temperature abuse during milk creaming and storage is decisive in the control of pathogenic microorganisms [41]. In a model-scale productions of Grana cheese artificially contaminated with high amounts (approx. 10^4^ cfu/mL) of *E. coli* O157:H7, *L. monocytogenes*, *Salmonella typhimurium*, and *St. aureus*, the thermal stress (e.g., the curd cooking and molding at temperature > 50 °C) in the technology of production was shown to be only partially effective in the control of the selected pathogens. Authors concluded that the barriers included in the whole technology of production would further contribute to ensure the safety of GP cheese [42]. A challenge test to evaluate the survival of *Mycobacterium avium* subsp. *paratuberculosis* (MAP) during the production process of PR and GP showed that the long ripening period leads to the total inactivation of the pathogen [43].

In starter cultures, the Qualified Presumption of Safety (QPS) approach includes the evaluation of each strain for the presence of acquired (and possibly transmissible) resistance to antibiotics. If this need is easily satisfied for selected starters, composed of a defined number of species and strains, it becomes problematic if undefined cultures, such as SN for GP, composed of an unknown number of strains in spontaneous, daily rotation are considered. In such case, the antibiotic resistance should be evaluated for each culture component. The susceptibility to several antibiotics, including penicillin G, ampicillin, vancomycin (van), gentamicin (gm), tetracycline (tet), and erythromycin (em), was tested for 141 LAB strains belonging to *L. helveticus*, *L. delbrueckii*, *Lcb. casei*, and *Lcb. rhamnosus* and isolated from SNs and ripened GP and PR cheeses. Cheese isolates were generally more resistant than isolates from NS, although no indication on genetic transferability of resistance was provided [44]. A QPS approach was applied to 32 dominant strains belonging to *L. helveticus*, *L. delbrueckii* subsp. *lactis*, *S. thermophilus*, and *Lim. fermentum* and isolated from SNs for GP. All *L. helveticus*, *L. delbrueckii* subsp. *lactis*, and *S. thermophilus* isolates were susceptible to van, gm, tet, and em, whereas two *Lim. fermentum* strains showed resistance to tet and gm; however, genes involved in lateral transfer of tet and gm resistances were absent in both strains [13].

Spoilage bacteria, such as butyiric clostridia and dairy propionibacteria, can be found in GP. With approximately 2% of cheese forms which are affected, the ‘late blowing’ is still a widespread problem in GP. A high load of spores in milk, associated with long periods of ripening leading to a significant decrease of the redox potential, facilitate an uncontrolled germination of the spores. Butyric clostridia are strict anaerobic microorganisms which, by consuming lactate or the residual lactose, carry out the butyric fermentation with accumulation of butyric acid, acetic acid, and high quantities of gases (CO_2_ and H_2_) which cause an irregular eye formation, slits, and off-flavors during ripening. Clostridial species and the number of spores involved in the late blowing may vary considerably among different cheese types. *Clostridium tyrobutyricum* (the main blowing agent and the most frequent isolated species), *Cl. sporogenes*, *Cl. butyricum*, and *Cl. beijerinckii* are the species most often detected in spoiled GP cheeses [45,46,47,48]. There are different approaches (bactofugation of milk, application of lysozyme and protective cultures) to limit the onset of this defect, with the consequent economic damage, in GP and similar hard cheeses [49]. However, the control of the number of butyric clostridial spores in milk remains the preferred option. Within the genus *Propionibacterium*, two main groups are recognized, related to their respective habitats: the “cutaneous”, isolated from human or animal skin and of clinical-health care interest, and the “dairy” or classical propionic acid bacteria (PAB), commonly found in milk and derivatives. The PAB species of dairy interest are *Propionibacterium freudenreichii* with its three subspecies ‘freudenreichii’ (lactose-negative), ‘shermanii” (lactose-positive), and ‘globosum’, *Prop. thönii*, *Prop. jensenii*, *Prop. acidipropionici*, *Prop. cyclohexanicum*, and *Prop. microaerophilum* [50]. *Propionibacterium freudenreichii* was detected in Grana Trentino cheese following sequencing of the PCR-DGGE bands and, more recently, in 115 GP samples covering the whole cheese production area by sequencing the 16S rRNA gene (DNA metabarcoding), resulting among the first 15 dominant taxa [31,32]. The presence of dairy PAB can be associated with cheese defects. At low numbers they may contribute to cheese aroma development while, at high concentration, they may be involved in the generation of the late blowing defect in GP and similar cheeses by converting lactic acid into propionic acid, acetic acid, and CO_2_ [51]. To avoid their excessive growth and consequent late blowing, the number of dairy PAB should already be limited in raw milk and further controlled in cheese, especially between 30 and 60 days of ripening [52].

## 5. Emerging Trends and Final Remarks

The microbiota of GP cheese is the result of the interaction between the microorganisms present in raw milk and those added with the SN culture, in turn modulated by the technology and cheese ageing conditions. The peculiar phases of production, as well as the long ripening time, lead to a precise dynamic of microbial populations which, following the autolysis determined by the extremely selective processing and maturation conditions, contributes significantly to the definition of the appreciated sensorial and nutritional qualities of the cheese. The introduction in recent years of increasingly sensitive and informative molecular analysis techniques made it possible to obtain an in-depth picture of the diversity and dynamics of the GP microbiota. This could allow on the one hand to better target and control the development of the useful microorganisms and, on the other hand, to evaluate the presence, in a viable (and possibly culturable) state, of species of functional importance [53]. This latter aspect could open future perspectives for the targeted isolation of strains of potential industrial interest.
